# Bone Status at Mandibular Condylar Fracture Osteosynthesis Plate After Healing Period

**DOI:** 10.3390/jfb17010031

**Published:** 2026-01-06

**Authors:** Izabela Gabryelczak, Marcin Kozakiewicz

**Affiliations:** Department of Maxillofacial Surgery, Medical University of Lodz, 251 Pomorska St., 92-213 Lodz, Poland; marcin.kozakiewicz@umed.lodz.pl

**Keywords:** processus condylaris mandibulae, fracture, surgical treatment, open rigid internal fixation, osteosynthesis, plate, screw, complication, bone resorption

## Abstract

Objectives: Against common belief, mandibular condyle fractures are not that rare, with morbidity rates ranging from 19 to 52%, depending on actual literature sources. Practitioners try to improve the surgical techniques applied to obtain the most satisfactory anatomical and functional effect. The purpose of this study is to identify the relationships that affect bone loss around screws and plates in mandibular condylar process fractures treated surgically using the Open Reduction and Internal Fixation (ORIF) technique. Materials and Methods: Our research covered 276 fractures of the base, low and high neck of the condylar process. No formal sample size calculation was performed; the study enrolled patients treated at the Department during the last 4 years, based on informed consent granted both prospectively and retrospectively compares to the actual treatment time. The study group was selected based on injury type and the ability to implement surgical treatment. The imaging modality selected for the study was computed tomography (CT), which was assessed in each case by the same operator. Therefore, there was no need to account for inter-rater variability in the results. Based on CT scans, we studied various parameters, including bone healing in the fracture area, bone loss in the screws and plates area, change in the length of the mandibular ramus following osteosynthesis and on the opposite side after 12 months, as well as deformities of the mandibular head. Results: Using screws and plates is the gold standard for treating mandibular condylar process fractures. The number of screws used affects the bone loss ratio around head of the screw. Another factor that impacts the results achieved is the distance from the plate edge to the lateral pole of the mandibular head. Statistical evaluation indicated that proximity to the lateral pole is a risk factor for bone resorption at the plate edge. Conclusions: Based on the collected data, it is possible to predict bone loss, determine the location and selection of plates and screws, as well as to plan the procedure, achieving the lowest possible loss rates.

## 1. Introduction

Against common belief, mandibular condyle fractures are not that rare, with morbidity rates ranging up to 52%, depending on actual literature sources [1,2,3]. Over the years, there has been a lot of debate about the method that yields better treatment result: surgical or conservative treatment. Wide-spread access to computed tomography and higher awareness of complications that result from the failure to treat this kind of trauma have led to more and more hospitals opting for surgical treatment of such injuries. Achieving proper stabilization through osteosynthesis allows better temporomandibular joint (TMJ) [4] function to be achieved. Osteosynthesis and the closed approach lead to some bone height loss, reaching 0.3–1.6 mm and 6.9 mm, respectively. Though evidence is limited, TMJ functional capacity restoration achieved following osteosynthesis, which ranges from 63 to 90%. This contrasts with the poor TMJ functional capacity restoration ratios achieved with closed treatment (40%) [3]. In light of this research, striving for more frequent application of surgical techniques that decrease bone resorption seems warranted.

Each medical center strives to improve the surgical techniques applied and obtain the most satisfactory anatomical and functional effect. There are numerous papers that explore attempts at identifying the best possible access technique, material and shape of plates used for osteosynthesis [5,6,7].

Titanium alloy is a material commonly used for fixations, as its biological interaction profile [8,9] allows for its removal after achieving bone union. Obtaining surgical access [10] to the condylar process is however associated with a number of anatomical difficulties. These usually result in the need for revision surgery within the area of the pre-existing scar. These procedures are often technically demanding, with synechiae that prevent easy access to the anatomical structures constituting one of the contributing factors. This anatomical region also requires the surgeon to work in the direct vicinity of the facial nerve and since scarring reduces visibility and hinders proper identification of anatomical structures, nerve injury is more frequent. This is further complicated by the patients’ reluctance for revision surgery, which they consider unnecessary. Where titanium plates and screws are left inside the body, some patients develop complications, as the material provokes resorption of the surrounding bone. This has been already reported for lag screw fixations [11,12,13], however data is lacking for miniplates used for osteosynthesis in this anatomical region.

Condylar process fractures have constituted the core work of our maxillofacial surgery department, leading to numerous papers being published, focused on bone loss around the screws used for osteosynthesis of high fractures, such as mandibular head fractures. There are limited references that would delve into the issue of post-healing bone loss seen around plates used for mandibular condylar fracture osteosynthesis.

With this in mind, relevant research would pave the way towards improving surgical techniques and allowing to better forecast cases where osteosynthesis plate removal would be required. The purpose of this study is to determine the extent of bone resorption related to fixation material remaining in the body after surgical treatment of extracapsular fractures of the mandibular condylar process.

## 2. Materials and Methods

### 2.1. Study Design and Ethical Approval

This retrospective study was conducted at the Department of Maxillofacial Surgery, Medical University of Lodz, Poland, and was approved by the institutional bioethics committee (RNN/125/15/KEW and RNN/738/12/KB). The study was carried out in accordance with the Declaration of Helsinki. Both prospectively and retrospectively treated patients were included based on written informed consent.

### 2.2. Study Population

The study population comprised 220 patients with a total of 276 fractures involving the base, low neck, or high neck of the mandibular condylar process. Patients were eligible if they had sustained extracapsular condylar fractures treated surgically using open reduction and internal fixation (ORIF).

Exclusion criteria included:

- Isolated condylar fractures treated conservatively or with screw fixation only;
- Incomplete radiological documentation;
- Lack of 12-month postoperative follow-up.

Fracture type and displacement were classified according to Kozakiewicz’s classification [14]. Patient histories included trauma mechanism, side of injury (unilateral/bilateral), concomitant mandibular or craniofacial fractures, and general trauma history. The most common causes were battery, falls, and bicycle or scooter accidents.

### 2.3. Surgical Procedure

**Figure 1:**
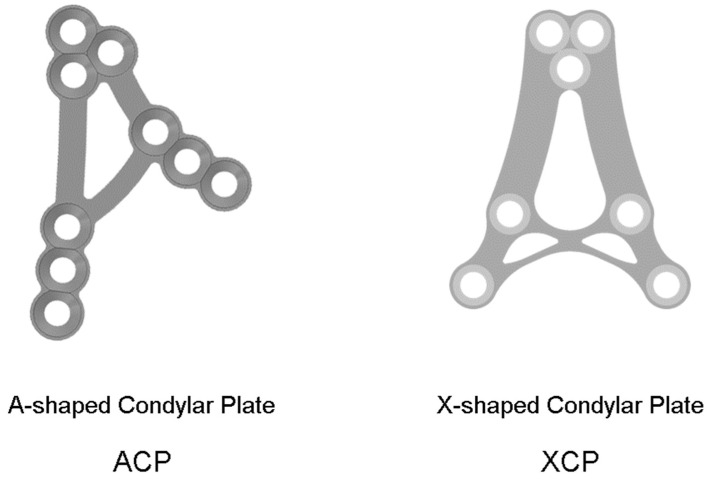
Rigid internal fixation was performed using the open approach and dedicated condylar ACP and XCP plates.

All patients were treated under general anesthesia with nasotracheal intubation. Open reduction and internal fixation were performed using ACP and XCP miniplates (grade 5 titanium, ChM, Lewickie, Poland) and 2.0 mm self-tapping screws (Figure 1).

The approach (preauricular or retromandibular) was selected based on fracture location and surgeon preference. For each case, the following intraoperative data were recorded:

- Time from injury to surgery;
- Duration of the procedurę;
- Number and length of screws;
- Type of osteosynthesis material.

All patients underwent standardized follow-up and postoperative CT imaging at 12 months.

### 2.4. Imaging and Measurement Protocol

Computed tomography (CT) was the imaging modality of choice. All scans were analyzed using RadiAnt DICOM Viewer (Medixant, Poznan, Poland; www.radiantviewer.com, accessed on 1 October 2022) by the same operator, eliminating inter-observer variability.

Evaluation was performed in the bone window (window level = 300 HU; window width = 1500 HU). All measurements were repeated twice by the same operator to ensure consistency. 

The following parameters were assessed:

- Consolidation in the fracture area;
- Bone loss around screws and plates;
- Change in mandibular ramus height after 12 months (operated and contralateral sides);
- Deformities of the mandibular head.

**Figure 2:**
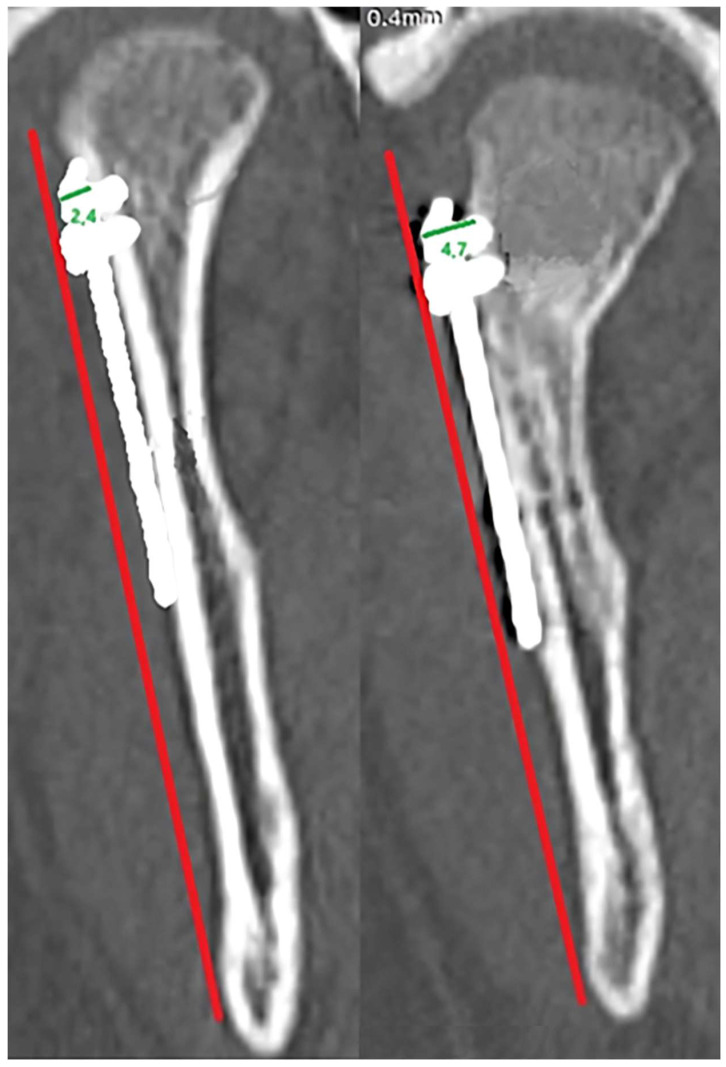
Measurement of bone loss around the screw head on CT images using the long axis of the bone as a reference line (red line). The measurement (green line) was taken as the perpendicular distance (mm) between the outer contour of the head and the edge of the bone.

Bone loss measurement Figure 2.

For each screw, the distance from the screw head to the bone border was measured parallel to the screw axis, immediately postoperatively and at 12-month follow-up. When resorption was observed around several screws, the mean value per plate was calculated. Measurements were taken in the frontal projection. A reference line perpendicular to the posterior border of the mandible was used to standardize orientation between scans.

### 2.5. Statistical Analysis

Statistical analysis included assessment of variable distribution and regression analysis to determine relationships between quantitative variables. Results were considered statistically significant at *p* < 0.05. All analyses were performed using Statgraphics Centurion XVI (StarPoint Technologies Inc., The Plains, VA, USA).

## 3. Results

The most common cause of mandibular condyle fracture was battery and falling from a height equal to at least the patient’s own height, as well as bicycle or scooter accidents. The amount of peri-screw bone resorption does not depend on the reason behind the injury (Table 1).

The average age of the treated patients was 34.5 ± 15 years, with 18.8% of the subjects female and 81.2% male.

Greater bone loss (*p* < 0.05) was observed in bilateral fractures of the mandibular condyle (0.86 ± 1.00 mm) and in unilateral (0.33 ± 0.49 mm) fractures. However, as there is an accompanying fracture of the body of the mandible, it seems to have no effect on bone resorption at the condylar fixation (*p* = 0.548). This does not apply to cases with an accompanying zygoma fracture, where higher resorption rates are reported with condyle osteosynthesis (1.50 ± 00.00 mm; *p* < 0.05). The presence of an orbital or maxillary fracture does not impact resorption at the condylar fixation site being investigated.

A summary of the collected data is presented in the tables below (Table 2 and Table 3).

**Figure 3:**
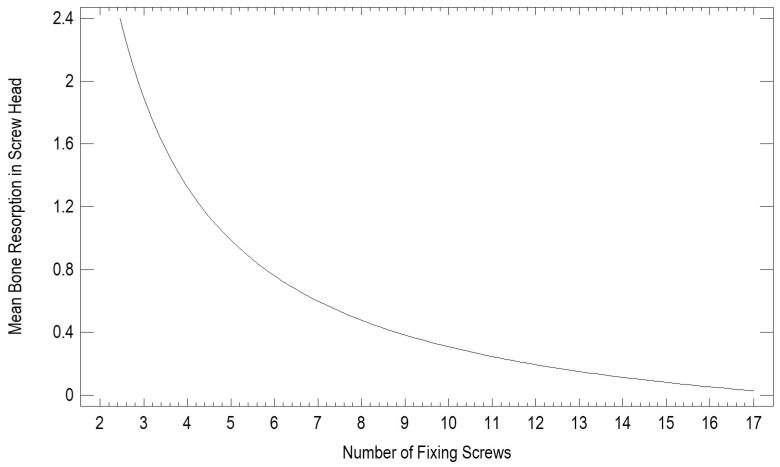
Our study recorded fixations performed using between 4 and 17 screws, revealing a statistically significant (*p* < 0.05) greater bone resorption at the screw heads (in mm) when the plate is fixed with a small number of screws (correlation coefficient −0.30).

Using screws and miniplates is the gold standard for treating mandibular condylar process fractures. The number of fixing screws used translates into differences in bone loss around the screw head. The more screws used, the less bone loss observed around the screw head (*p* < 0.05) (Figure 3).

**Figure 4:**
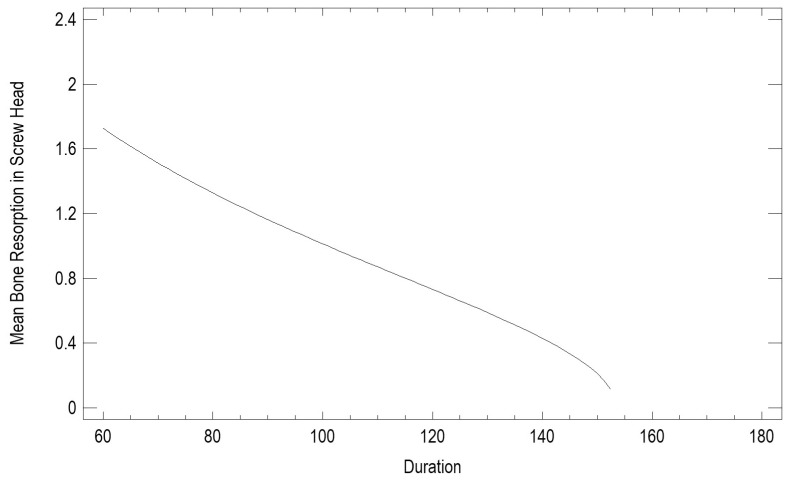
Relationship between bone resorption and surgical procedure duration.

Surgical procedure duration recorded ranged from 60 to 180 min. This variable was found to exert a moderately strong influence on treatment outcomes: short osteosynthesis duration (in minutes) led to greater resorption (in mm) in the screw head area (*p* < 0.0001, correlation coefficient 0.59) (Figure 4).

**Figure 5:**
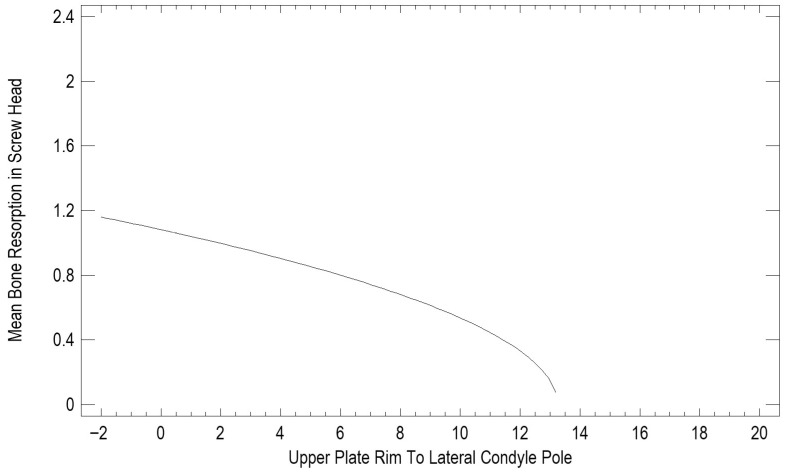
Bone resorption depends on the distance between the plate rim and the mandibular head lateral pole.

The impact of the distance from the plate rim to the mandibular head lateral pole (in mm) is presented in Figure 5. The values recorded ranged from those overlapping with the lateral pole (negative values) to a distance of as much as 20 mm. A statistical evaluation indicated that proximity to the lateral pole is a risk factor (*p* < 0.05) for bone resorption at the plate rim (Figure 5).

There was no relationship between resorption at the screw head and surgical treatment delay (*p* = 0.208), screw length used (*p* = 0.098), affected/intact ramus height ratio (*p* = 0.333). In contrast, a relationship was identified between the distance between the plate rim and the mandibular head lateral pole (*p* < 0.001), distance between the superior screw to the lateral pole (*p* < 0.0001), amount of resorption at the plate (*p* < 0.001), percentage of screw length resorbed (*p* < 0.0001), height of the ramus 12 months after treatment (*p* < 0.0001), as well as height of the ramus on the unaffected side after 12 months (*p* < 0.001), and the average resorption around the heads of the screws used.

There was no difference in the amount of bone resorption depending on the different fracture types: basal (0.48 ± 0.69 mm), low-neck (0.00 ± 0.00) and high-neck (1.14 ± 1.31 mm) The amount of resorption with screws is higher (1.12 ± 1.08 mm) in M-type fractures and lower (0.29 ± 0.45 mm) in *p*-type fractures (*p* < 0.01).

It was also noted that bone loss around the screw heads after mandibular condylar process ORIF is affected by the approach selected (*p* < 0.05). The largest loss was recorded with the preauricular approach, reaching approximately 0.74 mm.

Once the approach is chosen and the fracture is repositioned, fixing material selection is important. During the study, it was noted that the smallest average loss was recorded with XCP-type plates, reaching approximately 0.33 mm, straight plates yielded average bone loss of 0.37 mm and ACP plates led to the highest loss, reaching approximately 0.44 mm.

Interestingly, lack of visible fracture consolidation was not associated with increased bone loss around the screw heads. Postoperatively, patients had their occlusion examined, which revealed that it had no impact on bone loss (*p* = 0.46). Our data indicated that the greatest bone loss was seen in patients who did not achieve stable occlusion conditions due to a small number of teeth and not using dentures (*p* < 0.05).

**Figure 6:**
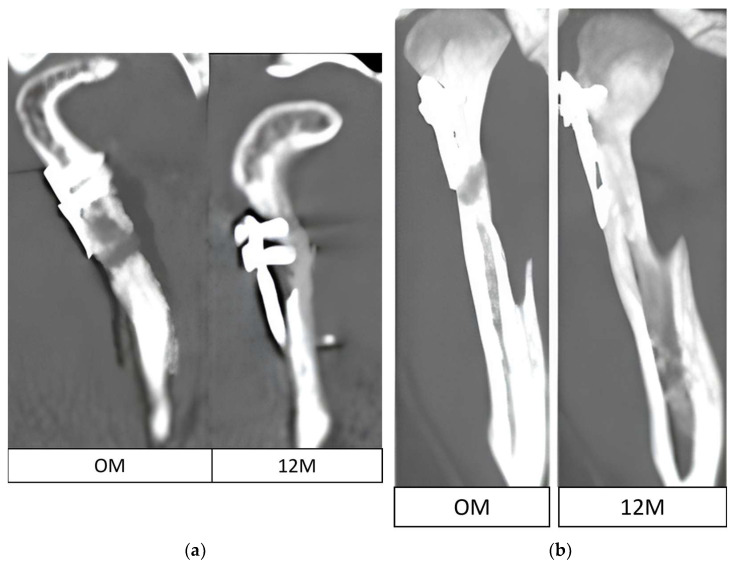
(**a**) Bone loss at the screw heads due to the plate being placed too close to the lateral pole (**b**) Bone loss at the lateral pole due to inserting the plate too close.

It was also noted that in the absence of osteosynthesis there is less atrophy at the screw heads (Figure 6).

## 4. Discussion

In line with previous results from other studies, we have identified that the most common reason for mandibular condyle fracture among women is falls, while in men the most common reason is battery. Moreover, females are at risk due to lower bone density [15]. Fortunately, these are low-energy injuries and generally women are a minority among patients treated for mandibular bone fractures.

A number of papers focus on bone loss around screws used for treating mandibular head fractures [4,16]. However, very few have discussed resorption around plates and screws used in lower condylar process fractures. Our findings regarding the relationship between screw number and bone loss correspond partly with those of Neff et al. [4], who also reported that increased fixation points improve load distribution and reduce cortical stress. Similarly, Kozakiewicz and co-workers [6] confirmed that plate design and screw configuration influence stability in condylar fractures. In our series, the general trend confirmed that a well-distributed fixation with multiple screws reduces localized bone remodeling, although the correlation remained weak, which aligns with prior biomechanical evidence.

Interestingly, a longer duration of osteosynthesis was associated with lower bone resorption around the screws, which seems counterintuitive. Usually, longer surgery indicates greater technical difficulty and may be seen as a negative factor [17]. However, in our study, longer procedures probably reflected more careful fracture reduction, more precise plate adaptation, or additional intraoperative verification, which could improve fixation stability and reduce stress on the surrounding bone. This observation contrasts with reports by Tatsumi et al. [2], where prolonged surgery correlated with increased complication rates. The difference may be due to the use of standardized protocols and stable fixation systems in our cohort.

Osteosynthesis location was found to be related to resorption extent. Based on our findings, factors that induce resorption are not limited to plate insertion on the articular surface or covering the lateral pole of the mandibular head. Even lower osteosyntheses within the mandibular neck entail resorption. Only distant fixation, more than 14 mm away from the lateral pole, ensures no bone resorption at the osteosynthesis site. This finding is consistent with the biomechanical conclusions of Pavlychuk et al. [18] and Kozakiewicz et al. [6], who emphasized the importance of avoiding fixation too close to the lateral pole to prevent stress concentration and plate-induced remodeling. These effects likely result from both local mechanical irritation and the shape of the fracture line rather than from the material itself. All of these factors do not affect the destruction of the mandibular head [19,20,21,22,23,24,25].

We observed that the impact of screw length used on bone loss extent is not considerable, therefore the surgeon should consider fixation stability, as well as bone amount and quality at the planned screw fixation site. Our findings suggest that local bone morphology may influence stress distribution and resorption tendencies, especially in patients with a shorter mandibular ramus.

More severe trauma along with technical difficulties resulting from lower fragments stability seem to explain the greater bone resorptions in bilateral condylar fractures. Greater bone loss is observed when accompanied by a fracture of the zygomatic bone. This observation parallels data from Skroch et al. [24], who also noted that combined midfacial trauma increases biomechanical stress on the condylar fixation area. An accompanying fracture of the mandibular body or other parts of the facial skeleton, except the zygomatic bone, do not impact bone resorption at the mandibular condyle osteosynthesis site. Given the known influence of bone metabolism on healing, postoperative optimization of calcium and vitamin D levels may be considered in future studies, but should not be inferred directly from the present data.

The finding that cases without full bone healing did not show significant bone loss may seem surprising. One possible explanation is that when the fracture is not completely united, the load is transferred through the fixation system, which keeps some mechanical stress on the bone around the screws and prevents atrophy. It may also mean that the bone in partially mobile fixation areas reacts less with remodeling. However, this result should be interpreted with caution, because a lack of consolidation usually indicates delayed or impaired healing. This contrasts with reports by Smolka et al. [25], who observed progressive bone loss in unstable or partially healed condylar fractures. The discrepancy may reflect differences in fixation rigidity and postoperative loading conditions.

Notably, the presented bone loss generally does not pose a threat to treatment outcomes, as it does not affect the loss mandibular ramus height. Exceptions here may include resorption observed at the upper rim of miniplates placed near the mandible head lateral pole. This may lead to involvement of the mandibular head in the resorptive process, shortening of the ramus and finally malocclusion. An exceptionally clinically devastating case may be seen in patients with significant bone resorption even on the lateral surface of the condylar process (away from the mandibular head), as this causes greater plate strain and may lead to plate fracture or pull-out of the fixation material due to exceeding the pull-out force for the residual screw fixation length (where the total length is usually only 6 mm). These results are in agreement with findings by Neff and Kolk [22], indicating that minor peri-screw bone remodeling does not compromise functional recovery when fixation remains stable.

## 5. Limitations

The main limitation of this study concerns the quality of the CT images presented in the manuscript, which may appear suboptimal due to the image sharing and publishing process. However, the original tomographic datasets used for quantitative measurements were of sufficient diagnostic quality and did not compromise the accuracy or reliability of the analysis. Additionally, this was a single-center study with a retrospective design, which may limit generalizability. Despite these constraints, the large number of analyzed cases and standardized imaging protocol strengthen the validity of the findings.

## 6. Conclusions

Bone resorption around fixation plates after mandibular condylar process osteosynthesis is influenced mainly by plate position and number of screws. Plates placed within 14 mm of the lateral condylar pole showed significantly greater peri-plate resorption, whereas using approximately nine screws minimized bone loss. These findings can guide surgical planning to improve long-term bone stability after condylar fracture fixation. Future studies should further explore biological contributors and refine optimal plate design.

## Figures and Tables

**Table 1:** Fracture etiology and bone resorption observed.

Etiology	Frequency %	Resorption mm
Bike accident	10	0.83 ± 0.68 ^1^
Car accident	6	0.00 ± 0.00
Interpersonal violence	41	0.28 ± 0.42
Fall (own body height)	19	0.19 ± 0.45
Fall (more than own body height)	12	1.14 ± 1.20 ^1^
Scooter accident	12	0.50 ± 0.53 ^1^

^1^ Normal distribution.

**Table 2:** Summary statistics.

Parameter	Mean ± SD	Median
Treatment delay [days]	10 ± 18	6
Surgery duration [min]	122 ± 29	120 ^1^
Number of fixing screws in plate	8.63 ± 1.64	9
Length of screws used [mm]	6.0 ± 1.0	6

^1^ Normal distribution.

**Table 3:** Resorption values recorded.

Parameter	Mean ± SD	Median
Maximal bone resorption around screw head [mm]	0.6 ± 0.9	0.0
Number of screw heads with resorption	0.8 ± 1.3	0.0
Mean bone resorption in screw head [mm]	0.4 ± 0.7	0.0
Distance from upper plate rim to lateral condyle pole [mm]	5.9 ± 4.9	5.0
Distance from upper plate rim/screw to condyle topc [mm]	15.0 ± 6.2	14.9
Highest screw bone resorption around screw head [mm]	0.6 ± 1.6	0.0
Bone resorption at the upper plate rim [mm]	0.6 ± 1.4	0.0

## Data Availability

The data on which this study is based will be made available upon request at https://www.researchgate.net/profile/Marcin-Kozakiewicz. accessed on 1 October 2024.
